# The parasite’s new clothes

**DOI:** 10.7554/eLife.15957

**Published:** 2016-04-15

**Authors:** Mark S Pearson, Alex Loukas

**Affiliations:** Australian Institute of Tropical Health and Medicine, James Cook University, Cairns, Australia; Australian Institute of Tropical Health and Medicine, James Cook University, Cairns, Australiaalex.loukas@jcu.edu.au

**Keywords:** Schistosoma mansoni, stem cells, parasitic diseases, Other

## Abstract

A population of stems cells continuously rejuvenates the outer surface of a human parasitic flatworm.

**Related research article** Collins JJ, Wendt GR, Iyer H, Newmark PA. 2016. Stem cell progeny contribute to the schistosome host-parasite interface. *eLife* **5**:e12473. doi: 10.7554/eLife.12473

**Image** The outer surface of an adult blood fluke is called the tegument

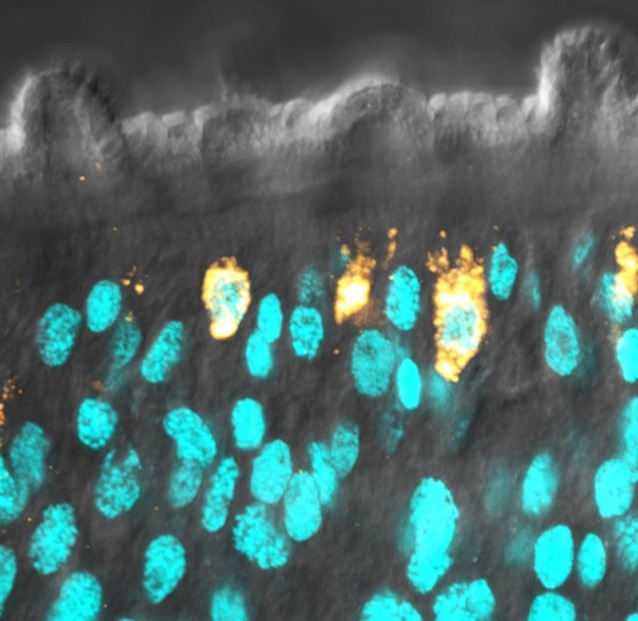

*The Emperor’s New Clothes* is a fairy tale about two cunning weavers who fool a vain emperor with a new “suit that is invisible to those who are unfit for their positions”. This tale, by Danish author Hans Christian Andersen, is strangely similar to a parasitic infection but with an important twist. Schistosomes are a type of parasitic flatworm, or fluke, and inhabit a site in the human body that is inhospitable to most other parasites – the blood. Yet instead of succumbing to our immune cells, the flukes can dwell in the bloodstream for many years. The fluke’s outer surface, or tegument, is key to its success as a long-lived parasite. The tegument clothes the entire adult fluke and hides its most vulnerable tissues from the prying eyes of the immune system. As such, it is us as human hosts (and not the flukes) who are fooled by the parasites’ remarkable “clothes”.

Schistosomes infect more than 200 million people in developing countries and cause a debilitating and neglected tropical disease called schistosomiasis, which kills about 300,000 people each year. Schistosomes are mostly unaffected by the onslaught of an infected person’s immune response (Cai et al., 2016). In fact, parasite biologists and immunologists have studied the “immuno-evasion tactics” of flukes for decades in an attempt to develop new drugs and vaccines to combat the infection.

A fluke’s tegument forms an almost impenetrable barrier between the parasite and its host. It also orchestrates an array of processes that allow the fluke to go about its parasitic lifestyle (Skelly and Wilson, 2006). As such, many researchers consider the tegument as a rich source of molecules that could be targeted by vaccines and drugs to combat schistosomiasis. Recent studies have revealed a great deal about the molecular composition of the tegument, but we know far less about how the tegument regenerates after it has been damaged (Figure 1). Now in eLife, James Collins, Phillip Newmark and colleagues from University of Illinois at Urbana-Champaign and UT Southwestern Medical Center tell a previously untold chapter in this tale of parasitic flatworms (Collins et al., 2016).

**Figure 1.**
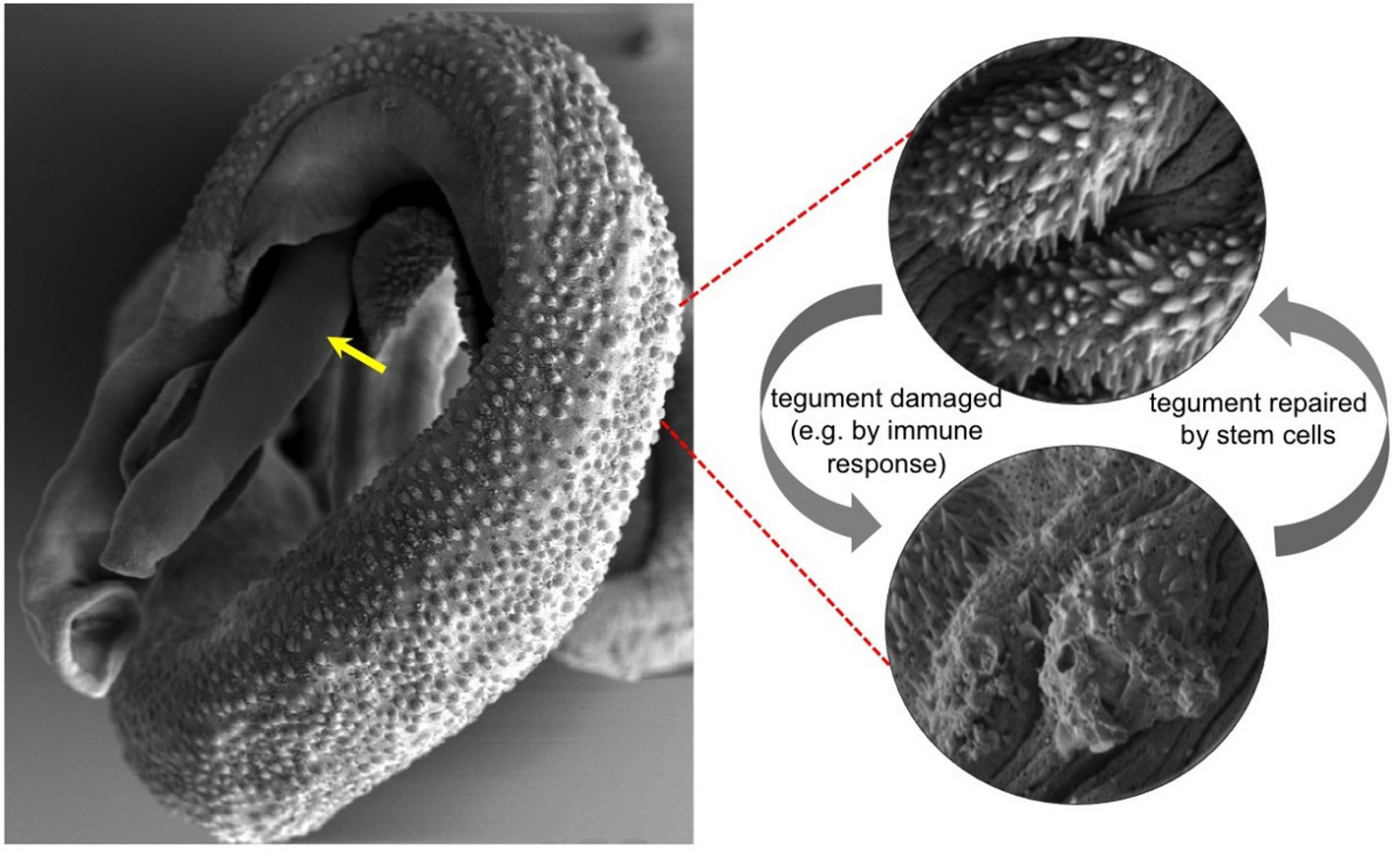
Electron micrograph of a pair of adult *Schistosoma mansoni* flukes. The male fluke is holding the female, and the front end of the female fluke (indicated with a yellow arrow) is protruding from the male. Red dashed lines show high magnification images of the outer surface, or tegument, of a healthy male fluke (top right) and a damaged male fluke (bottom right). Collins et al. revealed that stem cells called neoblasts provide the new cells needed to repair the tegument when it is damaged. Images courtesy of L. Becker, P. Wangchuk, J. Whan, M. Pearson and A. Loukas.

Collins, Newmark and colleagues had previously identified a group of stem cells – or neoblasts – in the parasitic flatworm *Schistomsoma mansoni* (Collins et al., 2013). Now they build on this discovery to show that these stem cells are biased towards generating new cells for the tegument and presumably replenish tissue at the host-parasite interface that has been damaged. Also, many of these stem cells contained tegument-specific proteins and appeared to be programmed to constantly rejuvenate the parasite’s outer surface (Collins et al., 2016).

Collins et al. depleted the schistosome’s stem cells by exposing them to a dose of radiation, and then looked at how gene expression in the flukes changed after two days (an early time point) and two weeks (a late time point). They reasoned that many genes that are active in stem cells would appear down-regulated at both early and late time points (because there are fewer stem cells left to express these genes). However, Collins et al. also inferred that genes that were down-regulated only at the later time point would instead most likely represent genes that are active in cells that rely on the stem cells in some way. For example, these genes might be active in tissues that must be replenished by new cells that develop from the stem cells.

To drill deeper into their gene expression dataset, Collins et al. then used gene silencing to eliminate proliferating stem cells. This allowed them to identify 105 genes that were not down-regulated at the early time point but were down-regulated at the late time point. They referred to these genes as “delayed irradiation sensitivity” (or DIS) genes. Many of the DIS genes encode for proteins that are expressed in the tegument (Braschi and Wilson, 2006). Indeed, many of the proteins encoded by DIS genes show promise as vaccines in animal models of schistosomiasis, and a small number of them are in early phase clinical development and trials (Hotez et al., 2010).

Collins et al. have shed light on one of the least illuminated yet critically important aspects of schistosome biology. Their work reveals how the schistosome tegument undergoes constant stem cell-mediated regeneration, a process that likely allows this highly evolved parasite to evade our best attempts to eradicate it. Future work might focus on methods by which to interrupt this regeneration process via drugs and/or vaccines. Indeed, praziquantel – a widely used drug for schistosomiasis – acts by destroying the tegument and revealing the vulnerable tissues underneath that can be attacked by the immune system in some individuals (Mutapi et al., 2013). Understanding the natural processes of tegument regeneration will accelerate the development of new control strategies that are urgently required for some of the world’s most impoverished and under-served populations.
